# Beyond Nutrient Profiling: Strengths, Limitations, and Emerging Challenges for Nutri-Score and Related Front-of-Pack Labeling Systems

**DOI:** 10.3390/nu18152422

**Published:** 2026-07-24

**Authors:** Jean Demarquoy

**Affiliations:** Université Bourgogne Europe, Institut Agro, INRAE, UMR PAM, 21000 Dijon, France; jean.demarquoy@u-bourgogne.fr

**Keywords:** nutri-score, nutrient profiling, front-of-pack labeling, food matrix, food processing, food quality, nutrition policy, dietary patterns

## Abstract

Front-of-pack nutrition labeling systems have become important tools for improving consumer understanding of nutritional information and supporting healthier food choices. Among these systems, Nutri-Score has emerged as one of the most widely implemented nutrient profiling models in Europe. Based on the Food Standards Agency Nutrient Profiling System, Nutri-Score summarizes selected nutritional characteristics of foods through a simplified five-level color-coded scale. A growing body of evidence indicates that Nutri-Score improves consumers’ ability to compare products, supports healthier purchasing decisions, and may encourage product reformulation. Epidemiological studies have reported associations between dietary patterns characterized by less favorable nutrient profile scores and increased risks of chronic diseases and mortality. Despite these strengths, important scientific and conceptual questions remain. This review examines the principles of nutrient profiling and assesses how effectively food quality can be represented by a limited set of nutritional criteria. Particular attention is given to nutrient reductionism, the use of the standardized 100 g reference, food matrix effects, the relationship between nutrient profiling and food processing, the influence of hedonic drivers and health claims on consumer behavior, and the growing importance of environmental contaminants as dimensions of food quality not captured by current algorithms. Future food evaluation systems may benefit from integrating complementary information related to food structure, processing characteristics, dietary context, and emerging insights from systems nutrition.

## 1. Introduction

### 1.1. General Overview

The increasing incidence and prevalence of obesity, type 2 diabetes, cardiovascular diseases, and other nutrition-related chronic diseases have intensified efforts to develop public health interventions that support healthier eating behaviors. Among these strategies, front-of-pack nutrition labeling systems have emerged as an important tool designed to provide consumers with simplified nutritional information and to encourage healthier purchasing decisions [1].

Over the last two decades, numerous nutrient profiling systems have been developed worldwide. These include the Multiple Traffic Light system in the United Kingdom, the Health Star Rating system in Australia and New Zealand, warning labels in several Latin American countries, and Nutri-Score in Europe [2]. Nutri-Score was officially adopted in France in 2017 and has subsequently been implemented or recommended in several European countries [3]. Recently, the U.S. Food and Drug Administration proposed a front-of-package nutrition labeling scheme known as the Nutrition Info box. Under this system, most packaged foods would be required to display simplified information on saturated fat, sodium, and added sugars, with each nutrient classified as Low, Med, or High. Unlike Nutri-Score, however, the proposed U.S. approach would not provide a single composite score or a color-coded overall assessment of nutritional quality. Instead, it would highlight selected nutrients of public health concern as a complement to the existing Nutrition Facts label [4].

Nutri-Score is derived from the Food Standards Agency Nutrient Profiling System (FSA-NPS), initially developed in the United Kingdom to regulate food advertising targeting children [5]. The algorithm allocates negative points for energy, sugars, saturated fatty acids, and sodium, while positive points are assigned for fiber, protein, and the content of fruits, vegetables, legumes, nuts, and selected vegetable oils. The resulting score is expressed per 100 g or 100 mL and converted into a five-category color-coded classification, with categories ranging from A (green) to E (red) according to the overall nutritional quality of the product [6].

Numerous studies have reported that Nutri-Score improves consumer understanding of nutritional information and may positively influence food choices [7]. Prospective cohort studies have reported that diets characterized by foods with more favorable FSAm-NPS scores, the nutrient profiling system underlying Nutri-Score, are associated with lower risks of cardiovascular diseases and all-cause mortality [8]. These findings have contributed to the increasing acceptance of nutrient profiling systems as public health tools.

Despite the widespread adoption of Nutri-Score and related nutrient profiling systems, several important conceptual and methodological questions remain unresolved. While these tools can simplify nutritional information and help consumers compare products, their underlying assumptions deserve critical examination. The aim of this narrative review is not to challenge the usefulness of Nutri-Score as a front-of-pack label, but rather to evaluate the scientific foundations on which it is built. This review aims to integrate evidence from nutrition science, food matrix research, food processing studies, consumer behavior, and food safety in order to critically examine the strengths and limitations of current nutrient profiling systems.

**Figure 1:**
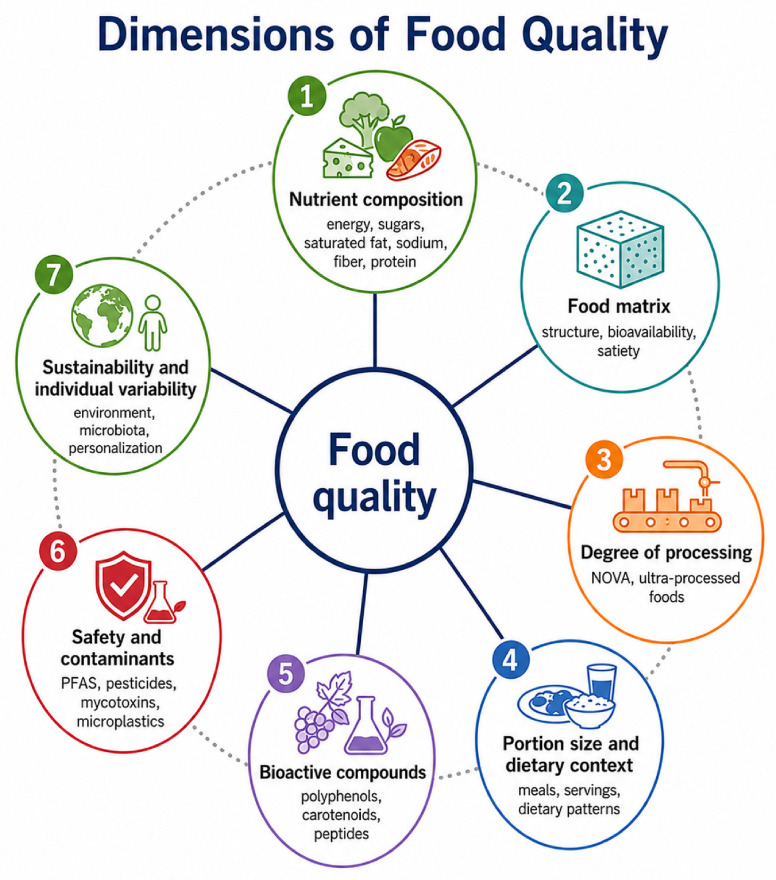
Multidimensional framework of food quality. Food quality is represented as the integration of seven complementary and interrelated dimensions: nutrient composition; food matrix; degree of processing; portion size and dietary context; bioactive compounds; safety and contaminants; and sustainability and individual variability. The solid lines connect each dimension to the central concept of food quality, while the dotted circle emphasizes that these dimensions interact and should not be assessed in isolation. The examples shown within each category are illustrative rather than exhaustive.

Taken together, these observations suggest that food quality cannot be adequately described by nutrient composition alone. Based on the principal domains recurrently identified in the literature reviewed, food quality is considered here as a multidimensional concept encompassing seven complementary dimensions: (1) nutrient composition, (2) food matrix, (3) degree of processing, (4) dietary context and portion size, (5) bioactive compounds, (6) food safety and contaminant exposure, and (7) broader contextual dimensions, including environmental sustainability and individual variability. This framework was developed as a pragmatic structure for the present narrative review and is not intended to represent a formally validated or exhaustive taxonomy (Figure 1).

Beyond these product-related dimensions, the reliability of producers and suppliers may also influence the consistency with which quality and safety standards are maintained over time. Indicators such as regulatory compliance records, traceability performance, continuity of certification, frequency and management of product recalls, and documented quality-control procedures could therefore provide a valuable complementary assessment of supply-chain integrity and long-term commitment to quality assurance. However, these indicators do not directly describe the nutritional, structural, toxicological, or environmental properties of the food itself. Producer reliability should consequently be regarded as a cross-cutting governance dimension that supports the credibility and durability of food-quality assessment rather than as an intrinsic characteristic of food quality. Section 2 and Section 3 provide the historical background and summarize the recognized strengths of nutrient profiling. The subsequent sections examine the dimensions insufficiently captured by current algorithms, while hedonic drivers and health-halo effects are treated as cross-cutting behavioral factors rather than intrinsic dimensions of food quality.

### 1.2. Methodology of the Review

This narrative review was based on searches of PubMed and Web of Science covering publications from January 2015 to July 2026. Search terms were used individually and in combination and included Nutri-Score, nutrient profiling, FSA-NPS, front-of-pack nutrition labelling, food matrix, food structure, food processing, NOVA classification, ultra-processed food, dietary patterns, portion size, consumer behavior, food contaminants, sustainability, systems nutrition, and precision nutrition. Priority was given to systematic reviews, meta-analyses, randomized or controlled studies, large prospective cohort studies, methodological papers, and recent authoritative reports. Earlier studies published before 2015 were retained when necessary to describe the historical development, conceptual foundations, or initial validation of nutrient profiling and front-of-pack labelling systems.

The database search was supplemented by backward and forward citation snowballing from key articles, systematic reviews, and methodological publications. Relevant grey literature, technical reports, guidance documents, scientific opinions, and policy documents were also identified through targeted searches of websites and document repositories of recognized international, European, and national organizations, including the World Health Organization, the Food and Agriculture Organization, the European Food Safety Authority, the European Commission, the Organization for Economic Co-operation and Development, the U.S. Food and Drug Administration, the UK Food Standards Agency, Santé publique France, and the French Agency for Food, Environmental and Occupational Health and Safety. Additional references were identified from the bibliographies of these reports and from documents cited in official recommendations and regulatory assessments. Publications were selected according to their relevance, scientific quality, recency, and contribution to the predefined conceptual domains of the review. Because this was a narrative rather than a systematic review, no formal quantitative synthesis, standardized risk-of-bias assessment, or certainty-of-evidence grading was performed.

The seven dimensions of food quality used to structure this review were identified through an iterative narrative synthesis of the principal domains recurrently discussed in the literature on nutrient profiling and contemporary food evaluation. They were selected because they describe complementary and partially independent aspects of food quality: nutrient composition, food matrix, degree of processing, portion size and dietary context, bioactive compounds, food safety and contaminants, and environmental sustainability and individual variability. This framework was developed specifically for this review. It was not based on a formal expert consensus, systematic evidence-mapping process, or validated classification method. It should therefore be viewed as a practical conceptual tool rather than a complete or universally accepted classification system.

## 2. The Evolution of Nutrient Profiling

### 2.1. Historical Development of Food Quality Assessment

The assessment of food quality has been a fundamental objective of nutrition science for decades. Historically, dietary recommendations were primarily developed to prevent nutrient deficiencies by ensuring adequate intakes of vitamins, minerals, proteins, and energy. Over time, nutritional science has evolved from a focus on deficiency diseases toward the prevention of chronic diseases and, more recently, the promotion of overall health and healthy dietary patterns [9]. During the second half of the twentieth century, accumulating evidence linking diet to chronic diseases highlighted the role of nutrients associated with adverse health outcomes, particularly saturated fatty acids, sodium, and added sugars [10] (Table 1).

This evolution led to the development of nutrient-based dietary recommendations and, subsequently, nutrient profiling systems designed to classify foods according to their nutritional composition. Nutrient profiling is the science of classifying or ranking foods based on selected nutritional criteria to support health promotion, disease prevention, and nutrition policy [11]. These systems have since become key tools in public health policy, food regulation, and front-of-pack nutrition labeling.

### 2.2. The SAIN/LIM Concept: A Multidimensional Vision of Food Quality

Among the various nutrient profiling approaches proposed during the early 2000s, the French SAIN/LIM system represented a good attempt to identify the multidimensional nature of food quality [12]. The system was developed in the context of efforts to establish nutrient profiling tools for public health policies and the regulation of health claims. The SAIN (Score of Nutritional Adequacy of Individual Foods) component evaluated the density of nutrients considered beneficial for health relative to energy content, whereas the LIM component quantified the presence of nutrients that should be limited, including sodium, saturated fatty acids, and added sugars. Importantly, these two dimensions were calculated independently rather than being merged into a single score. This dual approach recognized that foods may simultaneously contain desirable and undesirable nutritional characteristics. One of the main strengths of SAIN/LIM was its ability to preserve nutritional complexity by avoiding excessive aggregation of nutritional information. However, its complexity was considered a barrier to effective communication with consumers [13].

### 2.3. Development of the Food Standards Agency Nutrient Profiling System

At the same time, the United Kingdom developed the Food Standards Agency Nutrient Profiling System (FSA-NPS) [14]. The FSA-NPS introduced a simplified scoring framework in which foods received negative points for components associated with adverse health outcomes, including energy, total sugars, saturated fatty acids, and sodium. Positive points were assigned for fruits, vegetables, nuts, fiber, and protein.

Unlike SAIN/LIM, the FSA-NPS aggregated these positive and negative dimensions into a single numerical score. This simplification facilitated food classification and regulatory implementation while improving usability for both policymakers and consumers. Validation studies suggested that the FSA-NPS effectively differentiated foods according to their overall nutritional composition and showed reasonable agreement with dietary recommendations [5]. Consequently, the system became one of the most influential nutrient profiling models worldwide.

### 2.4. The Intended Goals of Nutri-Score

Before discussing the strengths and limitations of Nutri-Score, it is important to recall the objectives for which the system was originally developed. Nutri-Score was not designed to provide a comprehensive assessment of food quality, safety, processing level, or overall health impact. Rather, its primary purpose was to offer consumers a simple, standardized, and easily interpretable summary of selected nutritional characteristics at the point of purchase. By translating complex nutritional information into a color-coded scale, the system aims to facilitate product comparisons, encourage healthier food choices, and stimulate product reformulation. Consequently, the limitations discussed in this review should be interpreted in light of these original objectives and not as evidence that Nutri-Score fails to fulfill functions for which it was never intended. Nutri-Score was developed as a simplified graphical adaptation of the FSA-NPS algorithm [15]. These findings contributed to the adoption of Nutri-Score by France in 2017 and later by several other European countries (Table 2).

A recent assessment of the first decade of Nutri-Score implementation in Europe has also emphasized that the scientific evaluation of the label must be considered together with questions of harmonization, transparency, regulatory implementation, and communication at the European level [16].

### 2.5. Recent Revisions and Evolution of the Algorithm

An important characteristic of Nutri-Score is that its algorithm has not remained static since its implementation. Several expert committees have proposed revisions intended to improve consistency with national dietary recommendations and evolving scientific knowledge [17]. Recent modifications have affected the weighting of sugars, beverages, dairy products, whole-grain products, oils, and certain plant-based foods. These updates have led to the reclassification of numerous products and altered the relative ranking of several food categories.

From one perspective, these revisions demonstrate the capacity of the system to evolve in response to advances in nutritional science and changes in public health priorities. From another perspective, they highlight the intrinsic difficulty of summarizing food quality through a single numerical indicator. The need for repeated adjustments may also fuel concerns about the robustness and stability of food classifications, complicate comparisons across studies using different versions of the algorithm, and potentially weaken consumer confidence and understanding of the label. Indeed, because trust is a key determinant of consumer acceptance of front-of-pack labels, repeated modifications to food ratings may create uncertainty among consumers, particularly when the rationale for these changes is not clearly communicated [18].

## 3. Recognized Strengths of Nutri-Score

### 3.1. Improving Consumer Understanding of Nutritional Information

One of the major challenges in nutrition communication is the complexity of the information presented on food packaging. Conventional nutrition declaration tables provide detailed quantitative data on energy and nutrient content. However, their interpretation often requires the ability to identify and read information displayed in small print on food packaging, together with adequate nutritional literacy and numeracy skill and substantial cognitive effort. Many consumers have difficulty interpreting nutrition fact panels and identifying healthier options, particularly in time-constrained purchasing environments [19,20]. In response to these limitations, front-of-pack nutrition labels have been developed to provide simplified and readily interpretable nutritional information. The color-coded A-to-E format of Nutri-Score enables rapid product comparisons and reduces the cognitive burden associated with food choices at the point of purchase. Experimental and real-world studies have consistently shown that Nutri-Score improves consumers’ ability to rank foods according to their nutritional quality and facilitates healthier purchasing intentions and choices [21]. Importantly, these benefits appear particularly pronounced among individuals with lower levels of nutritional knowledge, education, and socioeconomic status, populations that are disproportionately affected by diet-related chronic diseases and health inequalities [22].

Nutri-Score may also have an educational effect beyond its immediate role in product comparison. By making nutritional quality more visible and easier to interpret, it may stimulate consumers’ curiosity about nutrition, encourage greater attention to dietary balance, and increase awareness of the relationship between food choices and health. In this way, Nutri-Score could contribute to a broader willingness to adopt healthier eating habits, although the extent of this effect is likely to vary according to individual knowledge, motivation, and socioeconomic context [23].

### 3.2. Standardization of Nutritional Information

Another important strength of Nutri-Score lies in its capacity to standardize the presentation of nutritional information. By applying a common algorithm across food products, Nutri-Score provides a consistent framework that allows consumers to compare foods within and between categories.

Standardization also facilitates communication by public health authorities and may contribute to harmonizing nutritional messages. Unlike individual nutrient claims, which frequently highlight only one favorable characteristic of a product, Nutri-Score attempts to provide an integrated assessment of several nutritional dimensions simultaneously [6]. Furthermore, the use of a standardized reference based on 100 g or 100 mL allows direct comparisons between products regardless of packaging size or serving suggestions. Although this approach has limitations that will be discussed later, it represents a methodological strength from a regulatory perspective because it relies on objective and reproducible criteria.

Although nutrient profiling systems were initially conceived as tools to support the regulation of nutrition and health claims, this objective has only been partially achieved. In practice, the correspondence between Nutri-Score ratings and the presence or relevance of nutrition and health claims remains limited. A favorable Nutri-Score does not predict the existence of authorized health claims, nor do products carrying such claims necessarily receive the highest Nutri-Score classifications. Consequently, Nutri-Score and health claims often provide complementary, rather than convergent, information to consumers [24]. This observation may appear somewhat counterintuitive, as both tools are generally expected to convey a consistent message about the healthfulness of a product.

### 3.3. Influence on Food Choice and Purchasing Behavior

A substantial body of experimental and observational research has investigated the effects of Nutri-Score on food selection. Randomized trials, online experiments, supermarket simulations, and field studies have generally reported that Nutri-Score improves the nutritional quality of food choices compared with the absence of labeling or alternative front-of-pack systems [25].

Meta-analyses and comparative studies suggest that Nutri-Score frequently outperforms other labeling formats in helping consumers correctly rank products according to their nutritional quality [26]. The visual simplicity of the system appears to facilitate rapid decision-making under real-world shopping conditions, where consumers often devote limited attention to nutritional information. Although the magnitude of these effects varies among studies and food categories, the overall evidence indicates that Nutri-Score can contribute to modest improvements in purchasing decisions at the population level [27].

A recent comprehensive review concluded that interpretive front-of-pack labels can improve the nutritional quality of food selection and purchasing, although effects vary according to label design, study setting, population characteristics, and the extent to which implementation is accompanied by broader public health measures [28].

### 3.4. Stimulating Product Reformulation

Beyond its effects on consumers, Nutri-Score has influenced food manufacturers. Because product classification directly determines the visual rating displayed on packaging, manufacturers may improve the nutritional composition of their products to achieve more favorable scores. Several studies have documented reductions in sodium, sugars, or saturated fatty acids following the implementation of nutrient profiling systems and front-of-pack labels [29]. Reformulation represents one of the most important potential public health benefits of nutrient profiling because it may improve the nutritional quality of the food supply independently of individual consumer behavior. From a regulatory perspective, reformulation can generate population-wide benefits without requiring continuous educational interventions. Even small improvements across large numbers of products may produce meaningful public health effects over time. A recent systematic review of 39 studies concluded that front-of-package labelling can influence product reformulation, although the magnitude and direction of changes vary according to the regulatory context, label format, food category, and study design [30].

Nevertheless, the desirability of such reformulation remains open to debate. Does adapting foods to meet the requirements of a nutrient profiling system truly lead to healthier products, or does it risk promoting a simplified and potentially misleading conception of food quality based primarily on a limited set of nutritional criteria?

### 3.5. Epidemiological Evidence Supporting Nutrient Profiling

The scientific legitimacy of Nutri-Score largely derives from studies evaluating the underlying FSA-NPS nutrient profiling model. Several large prospective cohort studies have reported associations between diets characterized by poorer nutrient profile scores and increased risks of obesity, cardiovascular disease, cancer, and all-cause mortality [31,32].

For example, analyses conducted within the European Prospective Investigation into Cancer and Nutrition (EPIC) cohort observed that individuals consuming diets composed of foods with less favorable nutrient profile scores exhibited higher risks of chronic disease and mortality [33]. Similar findings have been reported in French, British, and other European populations [31,34].

These observations suggest that the nutritional dimensions captured by nutrient profiling systems are relevant to health outcomes. The associations are biologically plausible, given the established roles of excess sodium, saturated fatty acids, sugars, and energy intake in the development of chronic diseases. However, it is important to recognize that these studies primarily evaluate associations rather than causality. Nutrient profiling systems were originally designed to rank foods according to selected nutritional criteria rather than to predict disease risk directly. Consequently, while epidemiological evidence supports the public health relevance of nutrient profiling, it does not necessarily demonstrate that food quality can be fully represented by a single score. An important distinction should nevertheless be made between efficacy and effectiveness. A substantial proportion of the evidence supporting Nutri-Score originates from experimental studies, online choice experiments, supermarket simulations, or controlled interventions in which consumers are explicitly exposed to the label. These studies generally demonstrate improvements in food ranking and purchasing intentions. However, demonstrating improvements in consumer choices does not necessarily imply measurable effects on obesity, cardiovascular disease, cancer incidence, or mortality at the population level. Such outcomes are influenced by numerous determinants including socioeconomic status, food availability, cultural habits, food prices, marketing, and overall dietary patterns. Consequently, while current evidence supports the usefulness of Nutri-Score as a public health tool, its long-term contribution to population health remains difficult to quantify directly and will likely depend on its integration with broader nutritional and public health policies.

A recent systematic review and meta-analysis of prospective cohort studies generally supported associations between less favorable dietary indices underpinning front-of-pack labels and increased risks of mortality, cardiovascular disease, and cancer. However, the pooled estimates were modest and accompanied by substantial heterogeneity, indicating that these indices should be interpreted as population-level indicators of dietary quality rather than as direct predictors of individual disease risk [35].

A summary of the principal evidence supporting Nutri-Score and related nutrient profiling systems is presented in Table 3.

## 4. Nutrient Reductionism: Can Food Quality Be Captured by a Limited Number of Nutrients?

### 4.1. The Nutrient-Centric Paradigm

For many years, nutrition science has focused primarily on individual nutrients rather than whole foods. This approach emerged during the twentieth century following major successes in identifying nutrient deficiencies and their prevention through the discovery of vitamins, minerals, and essential nutrients [10]. The nutrient paradigm subsequently expanded beyond deficiency diseases and became the dominant framework for understanding the relationships between diet and chronic disease. Nutritional recommendations increasingly focused on individual nutrients such as saturated fatty acids, sodium, sugars, dietary fiber, and cholesterol. This led to the development of nutrient profiling systems that classify foods based on their nutrient composition [36].

Nutri-Score represents a direct application of this paradigm. Its underlying assumption is that the health potential of foods can be adequately approximated by a limited number of nutritional variables. While this approach offers practical advantages for communication and regulation, its scientific validity has increasingly been questioned as nutrition science has evolved [37].

### 4.2. The Limited Number of Nutrients Included in Current Algorithms

The current Nutri-Score algorithm combines negative and positive points derived from a limited number of nutritional parameters [6,38]. These variables undoubtedly represent important determinants of nutritional quality and are supported by extensive epidemiological and clinical evidence. However, they capture only a fraction of the chemical and biological complexity of foods. Foods contain thousands of compounds that may influence health, including vitamins, minerals, trace elements, polyphenols, carotenoids, phytosterols, glucosinolates, bioactive peptides, fermentation-derived metabolites, and numerous other phytochemicals. Most of these compounds are absent from current nutrient profiling systems [39,40]. Consequently, foods with markedly different nutritional compositions may occasionally receive similar scores despite substantial differences in their content of potentially beneficial compounds. Because the algorithm relies on a restricted set of nutritional criteria, it does not capture the full complexity of food composition. Numerous constituents that may influence health, such as vitamins, minerals, polyphenols, carotenoids, and other bioactive compounds, are not considered. As a result, foods with distinct nutritional profiles and potential physiological effects may ultimately receive comparable classifications.

In addition, Nutri-Score does not consider antinutritional factors, naturally occurring toxins, process-induced contaminants, or environmental contaminants that may also influence the nutritional and toxicological characteristics of foods. Compounds such as phytates, oxalates, lectins, cyanogenic glycosides, mycotoxins, heavy metals, pesticide residues, acrylamide, and persistent environmental contaminants are not incorporated into the algorithm. Although many of these substances are naturally present, occur at concentrations below established safety thresholds, or are substantially reduced by food processing, they may nevertheless influence nutrient bioavailability, food safety, or long-term health outcomes [41]. Consequently, Nutri-Score evaluates only a limited subset of food characteristics and should not be regarded as a comprehensive indicator of overall food quality or safety.

### 4.3. Nutritional Components Omitted from Current Scoring Systems

#### 4.3.1. Vitamins and Minerals

Although micronutrients were historically at the center of nutrition science, they play only a limited role in most contemporary nutrient profiling systems. With the exception of indirect contributions through food categories such as fruits and vegetables, vitamins and minerals are not explicitly considered in Nutri-Score calculations. This omission may appear paradoxical given the essential physiological roles of micronutrients and their contribution to overall nutritional adequacy. Foods rich in calcium, magnesium, potassium, folate, vitamin B12, or vitamin D may not necessarily receive favorable scores if they simultaneously contain nutrients considered less desirable [42].

#### 4.3.2. Polyphenols and Phytochemicals

Over the past two decades, substantial evidence has accumulated regarding the biological activities of plant-derived phytochemicals. Polyphenols, flavonoids, anthocyanins, lignans, and numerous related compounds have been associated with antioxidant, anti-inflammatory, cardiometabolic, and microbiota-modulating effects [43]. These compounds contribute significantly to the health benefits associated with fruits, vegetables, legumes, tea, coffee, cocoa products, and extra-virgin olive oil. Yet, their presence is entirely absent from current nutrient profiling algorithms.

#### 4.3.3. Fermentation-Derived Compounds and Bioactive Peptides

Fermented foods provide another illustration of the limitations of nutrient-based food evaluation. Fermentation can generate numerous biologically active compounds, including peptides, organic acids, vitamins, and microbial metabolites that may influence host physiology [44]. Growing evidence suggests that fermented foods may exert health effects that cannot be fully explained by their nutrient composition alone. These dimensions remain largely invisible to current nutrient profiling approaches.

### 4.4. The Food Matrix Challenge

Perhaps the most important challenge to nutrient reductionism arises from the concept of the food matrix. The food matrix refers to the physical structure of foods and the complex interactions among nutrients and non-nutrient components within that structure [44,45]. Accumulating evidence indicates that the physiological effects of foods depend not only on nutrient composition but also on food structure, processing, texture, and nutrient interactions. The same quantity of nutrients may produce different metabolic responses depending on the matrix in which they are consumed [46]. Several examples illustrate this phenomenon. Dairy products often exhibit health effects that differ from those predicted solely by their saturated fat content [47]. Similarly, whole nuts show lower metabolizable energy values than expected because a fraction of their lipids remains trapped within intact cellular structures and escapes digestion [48,49].

These examples do not imply that food structure systematically overrides nutrient composition. Rather, they indicate that nutrient composition and matrix properties provide complementary information. Whereas some matrix effects are supported by controlled digestion and postprandial studies, their incorporation into general food scoring algorithms remains limited by the absence of standardized and readily measurable matrix indicators.

### 4.5. Foods as Complex Biological Systems Rather than Nutrient Carriers

The limitations of nutrient reductionism have led to alternative frameworks emphasizing food synergy and dietary patterns. According to this view, health effects result not only from individual nutrients but also from interactions among nutrients, bioactive compounds, food structures, and processing characteristics [37]. Consequently, food quality cannot always be predicted from nutrient composition alone. While nutrient profiling remains a valuable tool, it captures only one dimension of food quality. Future approaches may therefore need to integrate additional factors such as food matrix characteristics, processing level, and dietary context.

Taken together, the growing recognition of food complexity raises a fundamental question: can the health potential of foods be adequately represented by a limited set of nutrients, or does modern nutrition science require more holistic approaches to food evaluation?

## 5. Foods Are Consumed as Meals and Dietary Patterns Rather than as Isolated Products

Nutri-Score and related nutrient profiling systems are calculated at the level of individual food products. Their primary objective is to facilitate comparisons between foods available at the point of purchase by providing a standardized assessment of selected nutritional characteristics. However, an important conceptual distinction exists between the nutritional evaluation of individual foods and the assessment of diets as they are actually consumed.

In real-life settings, foods are rarely eaten in isolation. Most eating occasions involve combinations of foods that interact within meals and contribute collectively to overall dietary exposure. Consequently, the nutritional characteristics of a meal may differ substantially from those suggested by the classification of its individual components. Nutritional epidemiology has increasingly shifted its focus from individual nutrients and foods toward dietary patterns because health outcomes are influenced by cumulative dietary exposures and the interactions among foods consumed over time [37,50].

This distinction is particularly relevant when interpreting front-of-pack labeling systems. A favorable Nutri-Score indicates that a product possesses a relatively advantageous nutrient profile compared with alternative products within the same or related categories. However, it does not necessarily predict the nutritional quality of the meal in which that food will ultimately be incorporated. For example, whole-grain pasta may receive a favorable Nutri-Score owing to its fibre content and nutrient composition. Nevertheless, the nutritional characteristics of the final meal will depend on accompanying ingredients such as sauces, cheeses, processed meats, oils, or cream-based preparations. Similar considerations apply to breakfast cereals consumed with milk, breads consumed with spreads, or vegetables consumed with oils and dressings.

Beyond simple nutrient addition, foods may also interact biologically within meals. The bioavailability of micronutrients, glycemic responses, satiety signals, and postprandial metabolic effects may all be influenced by the composition of the meal as a whole rather than by individual foods considered separately. These interactions illustrate the broader concept that the physiological effects of foods are highly context-dependent.

Importantly, this limitation does not invalidate the usefulness of Nutri-Score. The system was designed to compare foods rather than meals or dietary patterns. Nevertheless, it highlights the distinction between product-level nutritional quality and overall dietary quality. Indeed, studies evaluating the Food Standards Agency nutrient profiling system from which Nutri-Score is derived have shown that meal-level and dietary-level nutrient profiling may provide information that differs from that obtained from isolated food classifications [51,52].

These observations suggest that nutrient profiling systems should be interpreted as tools for improving food selection within categories rather than as comprehensive indicators of meal quality or dietary healthfulness. Ultimately, health outcomes are determined by the overall composition of diets rather than by the nutritional characteristics of individual foods.

## 6. The Compensatory Nature of the Nutri-Score Algorithm

An important characteristic of the Food Standards Agency Nutrient Profiling System (FSA-NPS), which underlies Nutri-Score, is its compensatory scoring approach. The algorithm combines favorable and unfavorable nutritional attributes into a single numerical score. Positive points are assigned for dietary fiber, protein, and the proportion of fruits, vegetables, legumes, nuts, and selected vegetable oils, whereas negative points are assigned for energy density, sugars, saturated fatty acids, and sodium. The final classification therefore results from a balance between nutrients considered beneficial and those considered less desirable from a public health perspective.

This approach differs from earlier nutrient profiling systems such as the French SAIN/LIM model, in which favorable and unfavorable nutritional characteristics were evaluated separately rather than merged into a single score. The SAIN/LIM framework acknowledged that foods may simultaneously possess nutritional strengths and weaknesses. In contrast, the FSA-NPS was specifically designed to provide a simplified overall assessment that could be readily translated into a front-of-pack label and understood by consumers.

The compensatory approach offers several practical advantages. It facilitates product comparisons, reduces complexity, and allows multiple nutritional dimensions to be integrated into a single indicator. Furthermore, epidemiological studies have shown that dietary indices derived from the FSA-NPS are associated with risks of cardiovascular disease, cancer, and mortality, suggesting that the overall balance captured by the algorithm retains meaningful biological and public health relevance [26,27,28,29].

Nevertheless, the aggregation of distinct nutritional characteristics into a single score raises important conceptual questions. Nutrients do not exert interchangeable physiological effects. Dietary fibre, sodium, saturated fatty acids, sugars, vitamins, and bioactive compounds act through different biological pathways and influence health outcomes in different ways. Consequently, some authors have questioned whether favorable nutritional attributes should partially offset nutrients of concern within a single classification system.

Importantly, the existence of compensation should not be interpreted as evidence that the algorithm is fundamentally flawed. Indeed, any nutrient profiling system that seeks to summarize multiple nutritional dimensions into a single indicator inevitably requires some form of weighting and trade-off between favorable and unfavorable characteristics. The relevant scientific question is therefore not whether compensation should occur, but rather whether the chosen balance between positive and negative attributes adequately reflects current knowledge regarding diet and health.

Ultimately, the debate surrounding compensation illustrates a broader challenge in food evaluation. Simplicity and comprehensiveness are often competing objectives. The more information incorporated into a scoring system, the more difficult it becomes to communicate. Conversely, increasing simplicity inevitably requires some loss of nutritional detail. Nutri-Score represents one particular compromise between these competing objectives, and its strengths and limitations should be interpreted within this context.

## 7. The 100 g Reference Paradigm

### 7.1. Why Was the 100 g Reference Adopted?

Most nutrient profiling systems, including Nutri-Score, calculate food scores on the basis of 100 g or 100 mL of product. This approach was originally adopted to provide a standardized and objective framework for comparing foods independently of package size, serving recommendations, or individual consumption habits [14]. From both scientific and regulatory perspectives, the 100 g reference offers several advantages. It enables direct comparisons between products, ensures consistency across food categories and manufacturers, and facilitates the calculation of nutrient densities. In addition, nutritional information is commonly declared per 100 g or 100 mL in many jurisdictions, making this approach readily compatible with existing food labeling regulations. As a result, the standardized 100 g reference has become a cornerstone of most nutrient profiling systems and represents one of their principal methodological strengths.

### 7.2. The Disconnect Between Reference Quantities and Actual Consumption

**Figure 2:**
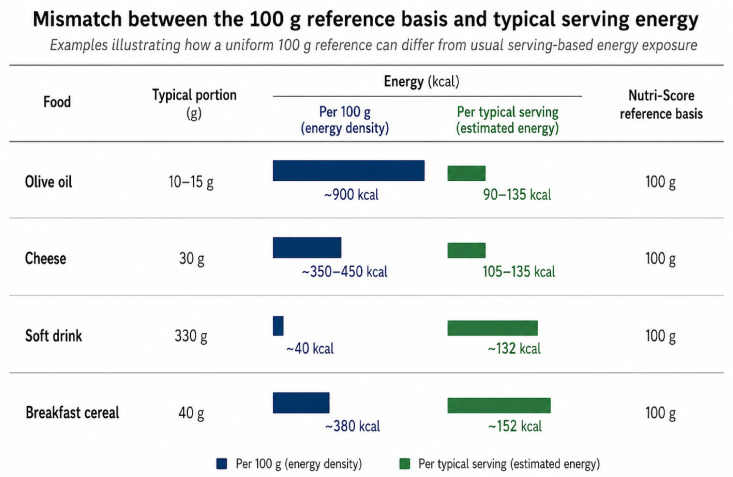
Comparison of energy density per 100 g with estimated energy intake per typical serving. Blue bars represent the energy content expressed per 100 g, whereas green bars show the approximate energy provided by a commonly consumed portion. The examples illustrate that the uniform 100 g reference basis used by Nutri-Score may differ substantially from actual serving-based energy exposure.

Despite its practical advantages, the 100-g reference may not always reflect real-world eating behaviors. Consumers do not eat nutrients in standardized quantities, nor do they consume all foods in 100 g portions (Figure 2). Foods differ considerably in their typical serving sizes. Some foods, such as beverages, yogurt, breakfast cereals, or pasta dishes, are frequently consumed in amounts exceeding 100 g. Conversely, oils, condiments, spices, and certain cheeses are generally consumed in much smaller quantities [53,54,55]. As a result, nutrient densities expressed per 100 g may not accurately represent actual nutritional exposure under normal dietary conditions. This discrepancy raises an important conceptual question: should food quality be assessed according to a fixed reference quantity or according to the quantities in which foods are actually consumed?

### 7.3. Olive Oil, Cheeses: Illustrative Examples

Olive oil and cheese are frequently cited as examples of the limitations of nutrient profiling systems. Extra-virgin olive oil, a cornerstone of the Mediterranean diet, has consistently been associated with favorable cardiovascular outcomes despite its high energy density (approximately 900 kcal/100 g), which inevitably influences nutrient-based classification [54,56]. Similarly, many cheeses receive less favorable ratings because of their saturated fat and sodium content, despite providing high-quality protein, calcium, vitamin B12, and bioactive compounds generated during fermentation [38,46]. Furthermore, cheeses are typically consumed in portions ranging from 20 to 40 g rather than 100 g. This discrepancy between reference quantities and actual serving sizes has fueled debates regarding the interpretation of nutrient profiling results for traditional foods. Importantly, the objective is not to argue that cheese should necessarily receive a higher rating. Rather, the example highlights the broader issue of whether a standardized reference can adequately capture the nutritional contribution of foods consumed in highly variable quantities.

Although recent revisions of the Nutri-Score algorithm have improved the ranking of olive oil relative to other added fats [57], both examples illustrate the limitations of evaluating foods using a standardized reference of 100 g. These foods are typically consumed in much smaller quantities, and nutrient profiles expressed per 100 g may therefore not accurately reflect actual dietary exposure or their contribution to overall dietary quality.

### 7.4. Portion-Based Alternatives

Several nutrient profiling systems have attempted to incorporate serving sizes or portion-based approaches. For example, some front-of-pack systems used in North America combine nutrient information with serving-based references. At first glance, portion-based approaches may appear more biologically relevant because they better reflect actual food consumption. However, they introduce additional challenges. Serving sizes are often poorly standardized across countries, food categories, and manufacturers. Industry-defined portions may vary substantially and can potentially be manipulated to improve nutritional profiles. Furthermore, portion sizes differ among individuals according to age, sex, cultural practices, and dietary habits [55].

### 7.5. Implications for Food Classification

The choice between 100 g and portion-based approaches ultimately reflects a trade-off between standardization and realism. The 100 g reference provides a robust and reproducible metric for comparing products but may occasionally generate classifications that appear inconsistent with common consumption patterns. This limitation is particularly evident for foods consumed in small quantities, foods used as ingredients, and foods whose health effects depend strongly on dietary context. Conversely, the use of portions introduces subjectivity and methodological complexity.

Rather than viewing these approaches as mutually exclusive, future nutrient profiling systems may benefit from integrating both perspectives. Food quality could potentially be evaluated using standardized nutrient densities while simultaneously providing information regarding realistic consumption scenarios.

## 8. Nutri-Score in the Context of Modern Nutrition Science

### 8.1. Nutrient Profiling Versus NOVA Classification

Over the last decade, the emergence of the NOVA classification system has profoundly influenced discussions regarding food quality and public health nutrition [58]. Unlike nutrient profiling systems such as Nutri-Score, which evaluate foods according to their nutritional composition, NOVA classifies foods according to the nature, extent, and purpose of industrial processing [58]. The two approaches are therefore based on fundamentally different scientific paradigms. Nutri-Score seeks to identify foods with favorable nutrient profiles, whereas NOVA attempts to characterize the degree of processing and industrial transformation. Importantly, these systems were not designed to measure the same dimensions of food quality. Consequently, disagreement between Nutri-Score and NOVA classifications should not necessarily be interpreted as evidence that one system is correct and the other is incorrect. Despite these limitations, these systems were not designed to measure the same dimensions of food quality. Consequently, disagreement between Nutri-Score and NOVA classifications should not necessarily be interpreted as evidence that one system is correct and the other is incorrect. Rather, such discrepancies suggest that food quality is multidimensional and cannot be fully captured by a single framework [59] (Table 4).

A recent scoping review identified substantial methodological variation in the identification of ultra-processed foods across studies, including differences in dietary assessment instruments, the availability of processing information, and the operational interpretation of NOVA criteria. These variations may result in exposure misclassification and limit comparisons between studies [60].

Although NOVA has been widely used in nutritional epidemiology, its application is not entirely free from methodological uncertainty. Classification may depend on the information available concerning ingredients, additives, formulation, and the purpose or extent of industrial processing. For composite foods and products with incomplete manufacturing information, different assessors may occasionally assign different NOVA categories. These reproducibility limitations should be distinguished from the epidemiological evidence associating high consumption of ultra-processed foods with adverse health outcomes. They nevertheless underline the need for clearer operational criteria, transparent classification procedures, and sensitivity analyses in studies using NOVA.

### 8.2. Favorably Scored Ultra-Processed Foods

One of the most frequently discussed criticisms of Nutri-Score concerns the classification of certain ultra-processed foods. Several highly processed breakfast cereals, plant-based products, low-sugar beverages, meal replacements, and reformulated foods may receive relatively favorable Nutri-Score ratings despite being classified as ultra-processed according to NOVA criteria [61]. These situations arise because Nutri-Score primarily evaluates nutrient composition. If a product contains limited amounts of sugars, saturated fatty acids, and sodium while providing fiber, protein, or plant ingredients, it may obtain a favorable score regardless of its degree of processing. For some researchers, such classifications represent a limitation because they may encourage consumption of industrially formulated foods whose long-term health effects remain incompletely understood [62]. Others argue that improving the nutritional composition of processed foods remains a legitimate public health objective and that nutrient quality should not be dismissed solely because of processing level [63]. The opposite situation may also occur. Certain minimally processed or traditional foods may receive less favorable Nutri-Score classifications despite limited processing. Examples frequently cited include some cheeses, cured meats, butter, and traditional regional products [64]. These foods may contain relatively high amounts of saturated fatty acids, sodium, or energy and therefore receive lower ratings despite their limited degree of industrial transformation. This divergence has generated considerable debate, particularly in countries with strong culinary traditions. However, it also illustrates a broader scientific issue: nutrient composition and degree of processing represent distinct characteristics that do not always correlate. A food can be minimally processed while possessing a nutrient profile considered less favorable according to current dietary recommendations.

Conversely, a highly processed food may exhibit a favorable nutrient profile after reformulation. This observation raises a broader question regarding front-of-pack nutrition labeling. Is it preferable to provide consumers with a single indicator intended to summarize the multiple dimensions of food quality, despite the difficulty of capturing such complexity within one score? Or should several complementary labels be displayed simultaneously, for example addressing nutrient profile, degree of processing, environmental impact, or other relevant characteristics? While a single label offers simplicity and ease of interpretation, it may inevitably overlook important aspects of food quality. Conversely, the use of multiple labels could provide a more comprehensive assessment but may also increase complexity and reduce consumer understanding. The challenge therefore lies in determining whether a sufficiently comprehensive and scientifically robust single indicator can realistically be developed, or whether food quality is inherently multidimensional and requires several complementary tools to be adequately communicated.

An additional limitation of nutrient-based scoring systems is that they assess the composition of the final product and are largely blind to the initial quality and processing history of the raw materials. Two products with similar final nutrient profiles may therefore receive the same score even when the freshness, microbiological history, traceability, or initial quality of their ingredients differ substantially. UHT milk provides a useful, although nuanced, illustration. UHT treatment produces commercially sterile milk, but it does not erase all consequences of poor raw-milk quality. Heat-stable proteases and lipases produced by psychrotrophic bacteria before processing may survive UHT treatment and subsequently contribute to bitterness, rancidity, sedimentation, and age gelation during storage. Advanced processing may therefore reduce visible or routinely measurable differences in the finished product, while upstream differences in raw-material quality remain undetected by nutrient profiling algorithms. Nevertheless, processing cannot be assumed to correct or conceal all microbiological or chemical defects. These considerations support the use of complementary information on raw-material specifications, traceability, processing history, and quality-control performance rather than their direct incorporation into a nutritional score.

### 8.3. Hedonic Drivers and Food Choice: Beyond Nutritional Quality

One of the major limitations of nutrient profiling systems is the implicit assumption that food choices are primarily driven by nutritional considerations. In reality, consumer behavior is influenced by a complex interplay of biological, psychological, social, cultural, and economic factors [65]. Among these determinants, hedonic properties play a central role. Taste, texture, aroma, appearance, and pleasure are often stronger predictors of food choice than nutritional information itself. Experimental studies consistently show that consumers prioritize sensory satisfaction when selecting foods. Foods rich in sugar, fat, salt, and flavor-enhancing compounds activate reward pathways that contribute to food preference and repeated consumption [66]. Consequently, the nutritional quality of a food does not necessarily determine its attractiveness or its actual consumption. A product receiving a favorable Nutri-Score may be ignored if it is perceived as less palatable, whereas a less favorably rated product may be frequently consumed because of its sensory appeal.

This distinction is particularly important because public health nutrition aims not only to classify foods but also to modify eating behaviors. Nutrient profiling systems evaluate nutritional composition but provide no information regarding palatability, reward value, satiety, or consumer acceptance. Future food evaluation frameworks may therefore benefit from acknowledging the central role of hedonic factors in shaping dietary behaviors [67].

### 8.4. Health Claims and the Risk of Nutritional Halo Effects

Food products are frequently marketed using nutrition and health claims highlighting specific beneficial characteristics. While such claims may provide useful information, they can also create a “health halo effect”, leading consumers to perceive products as healthier than they actually are [68,69]. For example, foods promoted as high in protein or rich in vitamins may still contain substantial amounts of sugars, saturated fats, sodium, or energy. Conversely, nutritionally favorable foods may carry no claims and receive less attention.

Nutri-Score was partly developed to provide a more global assessment of nutritional quality and to counterbalance the selective nature of individual claims. However, health claims and front-of-pack labels often coexist on the same product, sometimes conveying different messages. This situation illustrates the broader challenge of communicating food quality through simple indicators while avoiding oversimplification and marketing-driven distortions [70,71].

### 8.5. Beyond Nutrients: Environmental Contaminants and Emerging Food Safety Concerns

Nutrient profiling systems such as Nutri-Score were developed to evaluate selected nutritional characteristics of foods and were never intended to assess toxicological risks. Consequently, the absence of information regarding contaminants should not be interpreted as a flaw of the algorithm itself, but rather as a reflection of its original scope and objectives.

Nevertheless, contemporary discussions of food quality increasingly extend beyond nutritional composition alone. Food quality is determined not only by the nutrients a food contains but also by the substances to which consumers may be exposed through food consumption. Growing attention has therefore been directed toward contaminants such as pesticide residues, per- and polyfluoroalkyl substances (PFAS), heavy metals, microplastics, and processing-generated contaminants [72]. These compounds may influence health through mechanisms largely independent of nutrient composition, including endocrine disruption, oxidative stress, immune modulation, and chronic low-grade inflammation [73].

Importantly, there is currently no evidence that foods with favorable nutrient profiles are systematically associated with lower contaminant exposure, nor that foods with less favorable nutrient profiles are necessarily more contaminated. Nutritional quality and contaminant burden therefore represent largely independent dimensions of food quality. A product may receive an excellent nutrient profile score while also contributing substantially to exposure to specific contaminants, just as a food with a less favorable nutrient profile may contain relatively low contaminant levels.

Microplastics and PFAS illustrate this conceptual distinction. These contaminants have been detected in a wide range of foods, including drinking water, seafood, fruits, vegetables, salt, meat, eggs, and processed foods [74]. However, they are not considered by nutrient profiling systems because they represent toxicological rather than nutritional characteristics. This does not imply that nutrient profiling should incorporate contaminant measurements, which would be impractical given the geographic variability, analytical complexity, and temporal variability of food contamination. Instead, these examples emphasize that food quality is inherently multidimensional, encompassing not only nutritional composition but also food structure, processing, contaminant exposure, and environmental sustainability [75].

Recent systematic assessment confirms that microplastics have been reported in multiple food categories, while also identifying major heterogeneity and quality limitations in available dietary-exposure estimates. Consequently, current data support the relevance of microplastics as an emerging food-contaminant issue but do not yet allow robust comparisons of exposure between most food categories [76].

### 8.6. Food Quality: A Multidimensional Concept

The apparent contradictions between nutrient profiling systems and processing-based classifications may ultimately reflect the limitations of attempting to summarize food quality using a single dimension. Nutritional composition, food matrix characteristics, degree of processing, bioactive compounds, portion size, and dietary context each provide distinct information regarding the potential health effects of foods. Future food evaluation systems may therefore need to move beyond purely nutrient-based approaches and integrate multiple dimensions of food quality. Such developments could preserve the practical advantages of Nutri-Score while incorporating insights from contemporary nutrition science. Rather than asking whether Nutri-Score or NOVA provides the “correct” classification, the more relevant scientific question may be whether both systems capture complementary aspects of food quality that deserve consideration in future nutritional policies (Table 5). Recent proposals for multidimensional front-of-pack labelling have explored the joint communication of nutritional quality, processing characteristics, and environmental impact. These approaches illustrate the potential value of complementary information but also underline the risk of increasing cognitive complexity and obscuring the distinct scientific meaning of each dimension [77].

## 9. Future Directions

The evolution of Nutri-Score and other nutrient profiling systems reflects a broader transformation of nutrition science. Historically, food evaluation focused primarily on the identification of nutrients associated with deficiency diseases and, later, with chronic disease risk. Contemporary nutrition research increasingly recognizes that foods are complex biological systems whose health effects depend not only on nutrient composition but also on food structure, processing characteristics, bioactive compounds, consumption patterns, and interactions with individual physiology. Consequently, future nutrient profiling systems will likely face the challenge of reconciling scientific complexity with the need for clear and actionable consumer information (Table 6).

One important area of future development concerns the integration of food matrix effects. Growing evidence indicates that foods containing similar amounts of nutrients may exert different physiological effects depending on their physical structure, nutrient bioavailability, and interactions among food constituents. Dairy products, nuts, whole grains, and fermented foods provide well-known examples in which health effects cannot be fully predicted from nutrient composition alone [78]. Although current knowledge remains insufficient to allow routine incorporation of food matrix characteristics into nutrient profiling algorithms, advances in food science and systems nutrition may eventually permit more biologically relevant food classification approaches.

Food processing represents another important frontier. The increasing influence of the NOVA classification has highlighted the possibility that nutritional composition and degree of processing provide complementary information regarding food quality. However, processing encompasses a wide spectrum of technologies, ranging from simple preservation methods to highly engineered industrial formulations. Future systems may therefore need to move beyond simplistic distinctions between processed and unprocessed foods and develop more nuanced approaches capable of distinguishing beneficial, neutral, and potentially detrimental forms of processing.

The growing body of research devoted to bioactive compounds also challenges traditional nutrient-centered models. Polyphenols, carotenoids, phytosterols, glucosinolates, fermentation-derived metabolites, and numerous other food constituents have been associated with physiological effects extending beyond those explained by conventional nutrients. As analytical methods and food composition databases continue to improve, future profiling systems may progressively incorporate at least some of these components, allowing a more comprehensive assessment of food quality.

Technological developments may fundamentally transform how nutritional information is communicated. Front-of-pack labels are constrained by the need for simplicity and rapid interpretation. Digital technologies, including smartphone applications, QR codes, connected databases, and artificial intelligence-based decision-support tools, offer the possibility of providing consumers with access to far more detailed information than can be displayed on food packaging [79]. Such systems could simultaneously communicate nutrient composition, degree of processing, environmental impact, allergen content, bioactive compounds, and contaminant exposure while preserving the simplicity of front-of-pack labels for routine decision-making.

The emergence of precision nutrition raises additional questions regarding the future of food evaluation. Current nutrient profiling systems are designed for population-level recommendations and implicitly assume that foods exert broadly similar effects across individuals. However, increasing evidence suggests that dietary responses may vary according to genetics, age, physiological status, microbiota composition, metabolic health, and lifestyle factors. Although public health nutrition will continue to require generalizable recommendations, future digital tools may complement population-based nutrient profiling with more individualized assessments [80].

Recent systematic evidence suggests that personalized or precision-nutrition interventions may improve selected dietary and metabolic outcomes in individuals at elevated cardiometabolic risk. Nevertheless, heterogeneity in intervention design, personalization criteria, duration, and clinical endpoints currently limits their translation into standardized food-classification or front-of-pack labelling systems [81].

Environmental sustainability is also likely to become an increasingly important component of food evaluation frameworks. Food choices influence not only human health but also greenhouse gas emissions, biodiversity, water use, land occupation, and ecosystem integrity. Several initiatives have proposed combining nutritional and environmental indicators to guide food choices [82]. However, integrating these dimensions remains scientifically and ethically challenging because nutritional quality and environmental sustainability do not always align perfectly. Future policy discussions will need to address how these potentially competing objectives should be balanced.

Similarly, increasing attention to contaminants such as pesticide residues, PFAS, heavy metals, and microplastics highlights the emergence of food safety considerations that extend beyond traditional nutrition. Nutritional quality and contaminant burden represent largely independent dimensions of food quality, suggesting that future food evaluation systems may need to provide complementary information regarding both nutrition and toxicological exposure.

Nutrient profiling was initially developed not only to guide consumer choices but also to support regulatory decisions, particularly the use of nutrition and health claims. In the European Union, Regulation (EC) No. 1924/2006 envisaged the establishment of nutrient profiles to determine whether foods bearing nutrition or health claims should meet specific nutritional criteria before such claims could be made. However, despite this scientific framework, harmonized nutrient profiles have never been adopted at the European level. Nutri-Score represents a distinct application of nutrient profiling intended to provide consumers with a simplified front-of-package summary of nutritional quality rather than to regulate the authorization of nutrition or health claims [83]. Consequently, although both approaches rely on the principles of nutrient profiling, they differ in their objectives, regulatory status, and practical implementation, and there is currently no direct regulatory link between Nutri-Score and the authorization of health claims.

Front-of-pack and multidimensional labels may also be understood as a shared information channel between producers and consumers rather than solely as passive educational tools. By making selected and verifiable characteristics of food products more visible, such systems can allow producers to differentiate products according to nutritional composition, processing practices, traceability, environmental performance, or other quality-related attributes. Consumer preferences may then generate market feedback that encourages reformulation, improved quality control, greater transparency, and continuous improvement throughout the supply chain. In this respect, labeling may contribute to a positive feedback loop linking information disclosure, consumer trust, market differentiation, and producer incentives. However, this mechanism should not be assumed to operate automatically. Its effectiveness depends on consumer understanding, willingness to pay, retailer behavior, certification costs, regulatory credibility, and the absence of misleading claims. Multidimensional labeling should therefore be regarded as one component of a broader quality-governance system rather than as a sufficient guarantee of producer conduct or product superiority.

Ultimately, the future of food evaluation may not lie in the development of a single universal score capable of capturing all dimensions of food quality. Rather, it may involve integrated frameworks combining nutrient composition, food matrix characteristics, processing level, bioactive compounds, environmental sustainability, contaminant exposure, and dietary context. Whether these dimensions should be incorporated into a single composite indicator or communicated through multiple complementary tools remains an open scientific and regulatory question. Nevertheless, the trajectory of nutrition science suggests that future food classification systems will progressively evolve from nutrient-centered models toward more holistic representations of food quality that better reflect the biological complexity of foods and diets.

## 10. Conclusions

Front-of-pack nutrition labeling systems have become important components of public health strategies aimed at improving food choices and encouraging healthier food environments. Among these systems, Nutri-Score has emerged as one of the most widely implemented nutrient profiling models in Europe, owing largely to its simplicity and its capacity to translate complex nutritional information into a format that consumers can interpret rapidly. Available evidence indicates that Nutri-Score improves objective understanding of nutritional information, facilitates product comparisons, and may encourage healthier purchasing decisions and product reformulation, consistent with epidemiological associations between the underlying FSA-NPS algorithm and chronic disease outcomes.

Several of the limitations discussed in this review reflect long-standing and well-documented debates in the nutrition science literature. The tension between nutrient reductionism and the biological complexity of whole foods, the consequences of applying a standardized 100 g reference to foods consumed in highly variable quantities, and the divergence between nutrient-based classifications and processing-based frameworks such as NOVA have each been the subject of extensive prior discussion and dedicated empirical work. The purpose of this review is not to present novel criticisms, but to synthesize the available evidence and place it within a coherent framework for evaluating the current strengths, limitations, and future directions of nutrient profiling.

The contribution of this review lies less in any single critique and more in the attempt to situate these established debates alongside dimensions of food quality that are comparatively underexplored in the Nutri-Score literature specifically. Environmental contaminants, including PFAS and microplastics, are rarely discussed in the same analytic frame as nutrient profiling, yet they represent a dimension of food quality that operates entirely independently of nutrient composition and is likely to gain regulatory and scientific attention in coming years. Similarly, while hedonic drivers and health-halo effects are well studied in consumer behavior research, they are less frequently integrated into reviews that are primarily concerned with the algorithmic structure of nutrient profiling systems. By bringing nutrient composition, food matrix effects, processing classification, dietary context, consumer behavior, and contaminant exposure into a single conceptual framework, this review aims to clarify that these dimensions are not competing explanations for food quality but complementary and partially independent axes that current single-score systems were never designed to capture simultaneously.

This distinction matters for how the findings of this review should be used. The case for revisiting nutrient reductionism or the 100 g reference can draw on a substantial existing evidence base and does not require new justification here. The case for incorporating processing characteristics, hedonic factors, and contaminant exposure into future food evaluation frameworks is comparatively newer ground, and the recommendations offered in this regard should be read as a proposed direction for future research and policy discussion rather than as a synthesis of settled consensus.

Ultimately, the central question is not whether Nutri-Score is correct or not, but whether food quality can be adequately represented by a single score derived from a limited nutrient set. Current evidence suggests that nutrient profiling provides valuable and well-validated information, but that a comprehensive assessment of food quality requires integrating dimensions that extend beyond nutrient composition. The purpose of this review is not to present criticisms, but to synthesize the available evidence within a coherent framework for evaluating the current strengths, limitations, and future directions of nutrient profiling. More broadly, it aims to contribute to ongoing efforts to improve how nutritional quality is assessed, interpreted, and communicated to consumers, healthcare professionals, and policymakers.

## Figures and Tables

**Table 1:** Evolution of Food Quality Assessment.

Period	Main Concept of Food Quality	Assessment Criteria	Representative Examples	Limitations
Antiquity to 19th century	Food as nourishment and safety	Freshness, taste, appearance, absence of spoilage	Traditional food inspection, sensory evaluation	Subjective, no scientific basis
Early 20th century	Prevention of nutrient deficiencies	Essential vitamins, minerals, proteins, energy	Recommended dietary allowances, food composition tables	Focused on deficiency diseases rather than chronic diseases
1950s–1970s	Nutrient-based evaluation	Individual nutrients associated with health (fat, sugar, sodium, fiber)	Nutrition Facts labels, dietary recommendations	Foods considered as collections of isolated nutrients
1980s–1990s	Nutrient profiling	Simultaneous consideration of multiple nutrients	SAIN/LIM, first nutrient profiling models	Algorithms remained complex and not consumer-friendly
Late 1990s–2000s	Public health-oriented nutrient profiling	Positive and negative nutrient balance	FSA Nutrient Profiling System (FSA-NPS), health claim eligibility systems	Still largely nutrient-centric; food matrix poorly considered
2010s	Front-of-pack nutrition labeling	Simplified summary score based on nutrient profiling	Nutri-Score, Multiple Traffic Light, Health Star Rating	Simplification may obscure nutritional complexity
Late 2010s–2020s	Food processing and dietary patterns	Degree of processing and whole-diet quality	NOVA classification, Mediterranean Diet Score	Limited integration with nutrient profiling systems
Emerging approaches	Holistic food quality	Nutrient density, food matrix, bioavailability, sustainability, environmental impact, cultural aspects, personalization	Multi-dimensional food quality indices, precision nutrition, sustainable diet indices	No international consensus; difficult to summarize in a single score

Evolution of concepts and methods used to assess food quality, illustrating the transition from sensory evaluation and nutrient adequacy toward multidimensional approaches integrating nutrient density, food matrix, food processing, sustainability, and personalized nutrition.

**Table 2:** Historical Evolution of Food Quality Assessment.

Period	Main Concept of Food Quality	Assessment Criteria	Key Milestones and Representative Examples	Main Limitations
Mid-19th to early 20th century	Food purity and prevention of adulteration	Chemical composition, authenticity, contamination, fraudulent substitution	Development of analytical food chemistry and microscopy; UK Adulteration of Food and Drink Act of 1860; US Pure Food and Drugs Act of 1906	Focused mainly on fraud and acute safety rather than nutritional quality
1910s–1940s	Prevention of nutrient deficiencies	Vitamins, minerals, protein, and energy adequacy	Identification of vitamins; development of food composition tables; introduction of the first Recommended Dietary Allowances in the 1940s; food fortification programs	Primarily designed to prevent deficiency diseases
1980s–1990s	Standardized nutrition information and overall diet quality	Multiple nutrients, recommended intake levels, and dietary adequacy	Expansion of mandatory nutrition labelling; US Nutrition Labeling and Education Act of 1990; Diet Quality Index; Healthy Eating Index	Information remained complex and required substantial nutritional literacy
2010s	Interpretive front-of-pack nutrition labelling	Simplified nutrient profile communicated through colors, letters, stars, or warning symbols	Multiple Traffic Light label; Health Star Rating; Nutri-Score; nutrient warning labels in several countries	Simplification may obscure food complexity and differences between algorithms
Late 2000s–2020s	Food processing, food matrix, and dietary patterns	Degree of processing, food structure, ingredient formulation, and whole-diet quality	NOVA classification; Mediterranean Diet Score; increased attention to ultra-processed foods and food-matrix effects	Processing classifications may be difficult to apply consistently and do not always reflect nutrient quality
Late 2010s–2020s	Sustainability and planetary health	Nutritional quality, greenhouse-gas emissions, land and water use, biodiversity, and affordability	Sustainable diet indices; life-cycle assessment; planetary-health diet frameworks; environmental food labels	Environmental indicators vary substantially according to production methods and data sources
Emerging approaches	Multidimensional and personalized food quality	Nutrient density, bioavailability, food matrix, processing, contaminants, sustainability, cost, culture, and individual characteristics	Multidimensional food-quality models; precision nutrition; digital decision-support tools; microbiome- and metabolomics-informed approaches	No international consensus; data-intensive; difficult to communicate through a single score

Historical evolution of food quality assessment, illustrating the progressive shift from food purity and nutrient adequacy toward multidimensional concepts integrating processing, sustainability, and personalized nutrition.

**Table 3:** Summary of the evidence supporting Nutri-Score and related nutrient profiling systems.

Domain	Type of Evidence	Main Findings	Strength of Evidence	Representative References
Consumer understanding	Randomized controlled trials, experimental studies, comparative labeling studies	Nutri-Score consistently improves consumers’ ability to identify and rank foods according to nutritional quality compared with no label.	Strong	Egnell et al., 2019 [22]; Song et al., 2021 [26]; Crosetto et al., 2016 [21]
Food choice and purchasing intentions	Randomized trials, online experiments, supermarket simulations	Nutri-Score generally leads to the selection of products with more favorable nutrient profiles.	Moderate to strong	Egnell et al., 2021 [22]
Real-world purchasing behavior	Field studies and observational studies	Positive effects are observed under real shopping conditions, although the magnitude of change is generally modest and varies across populations.	Moderate	Cecchini and Warin, 2016 [1]
Product reformulation	Observational studies and industry analyses	The presence of front-of-pack labeling may encourage manufacturers to reduce sugar, sodium, and saturated fat content.	Moderate	Vyth et al., 2010 [29]
Overall dietary quality	Prospective cohort studies	Diets composed of foods with more favorable nutrient profile scores are generally associated with higher overall dietary quality.	Strong	Julia et al., 2015 [15]
Cardiovascular disease risk	Large prospective cohorts	Less favorable FSA-NPS dietary scores are associated with increased CV disease risk.	Moderate to strong	Deschasaux-Tanguy et al., 2024 [8]
Cancer risk	Prospective cohort studies	Associations have been reported between poorer nutrient profile scores and increased risk of several cancers.	Moderate	Donnenfeld et al., 2015 [34]
All-cause mortality	Large multicenter prospective cohorts	Less favorable nutrient profile scores are associated with higher mortality risk.	Moderate to strong	Deschasaux et al., 2020 [33]
Public health relevance	Integration of experimental, epidemiological, and policy evidence	Available evidence supports the use of nutrient profiling systems as useful public health tools, although they represent only one dimension of food quality.	Strong	EFSA, 2022 [11]; Hercberg et al., 2022 [6]

Comparison of major nutrient profiling systems, highlighting their scoring principles, intended applications, validation evidence, and principal strengths and limitations.

**Table 4:** Comparison of Major Food Quality Assessment and Front-of-Pack Labeling Systems.

Feature	Nutri-Score	NOVA	SAIN/LIM	Health Star Rating	FDA Nutrition Info box
**Primary objective**	Summarize nutritional quality	Classify foods by processing	Separate beneficial/limiting nutrients	Summarize nutritional quality	Highlight nutrients of concern
**Main criterion**	Selected nutrients	Degree of processing	Nutrient adequacy and nutrients to limit	Selected nutrients	Saturated fat, sodium, added sugars
**Overall score**	Yes (A–E)	No	No (dual scores)	Yes (0.5–5 stars)	No
**Color coding**	Yes	No	No	Yes	No
**Food processing considered**	No	Yes	No	Limited	No
**Food matrix considered**	No	No	No	No	No
**Bioactive compounds considered**	No	No	No	No	No
**Portion size considered**	No (100 g/100 mL)	No	No	Partly	Yes (serving-based)
**Primary use**	Consumer FOP label	Research and dietary guidance	Scientific/regulatory tool	Consumer FOP label	Consumer FOP label
**Main strength**	Simple and intuitive	Captures processing	Preserves nutritional complexity	Easy comparison	Simple nutrient-specific information
**Main limitation**	Nutrient reductionism	Processing only	Complex for consumers	Algorithm complexity	No overall nutritional assessment

Comparison of major food quality assessment and front-of-pack labeling systems according to their objectives, evaluation criteria, strengths, limitations, and coverage of key dimensions of food quality.

**Table 5:** Dimensions of Food Quality Considered by Nutri-Score and Complementary Dimensions Not Captured by Current Nutrient Profiling Systems.

Dimension of Food Quality	Considered by Nutri-Score	Examples	Potential Impact on Health
Macronutrient composition	✓	Energy, sugars, saturated fat, sodium	Cardiometabolic health
Beneficial nutrients	✓ (partially)	Fiber, protein	Metabolic health and satiety
Vitamins and minerals	✗	Calcium, vitamin D, potassium, folate	Nutrient adequacy and physiological functions
Bioactive compounds	✗	Polyphenols, carotenoids, phytosterols, glucosinolates	Antioxidant, anti-inflammatory and cardiometabolic effects
Food matrix	✗	Whole grains, dairy matrix, whole nuts	Bioavailability, digestion, satiety and metabolic responses
Degree of processing	✗	NOVA classification	Potential influence on chronic disease risk
Portion size and dietary context	✗	Olive oil, cheese, composite meals	Actual dietary exposure and meal quality
Hedonic characteristics	✗	Taste, texture, aroma	Food choice, food preference and adherence
Contaminants and food safety	✗	PFAS, microplastics, pesticides, mycotoxins	Toxicological risk and long-term health
Environmental sustainability	✗	Carbon footprint, biodiversity, water use	Planetary health and sustainable diets
Individual variability	✗	Genetics, microbiota, metabolic status	Precision nutrition and personalized dietary responses

✓ indicates that the criterion was considered, whereas ✗ indicates that it was not considered.

**Table 6:** Current Limitations of Nutrient Profiling Systems and Potential Future Developments.

Current Limitation	Scientific Rationale	Potential Future Development	Main Challenges
Limited number of nutrients	Food quality extends beyond a restricted set of nutrients.	Incorporate selected bioactive compounds and additional nutritional markers.	Limited food composition databases and uncertain weighting.
No consideration of the food matrix	Food structure influences digestion, nutrient bioavailability and metabolic responses.	Integrate food matrix characteristics into nutrient profiling.	Lack of standardized metrics.
No assessment of food processing	Processing may influence health independently of nutrient composition.	Combine nutrient profiling with processing-based classifications (e.g., NOVA).	Heterogeneity of processing technologies.
100 g reference basis	Standardized quantities may not reflect actual consumption.	Hybrid systems combining 100 g and realistic serving sizes.	Poor standardization of serving sizes.
No dietary context	Foods are consumed within meals and dietary patterns.	Meal- and diet-level nutrient profiling.	Greater complexity for consumers.
No consideration of contaminants	Food safety represents an independent dimension of food quality.	Complementary contaminant information via digital tools.	Large geographic and temporal variability.
Population-based approach	Individuals respond differently to the same foods.	Precision nutrition using genetics, microbiota and metabolic phenotyping.	Limited clinical validation and implementation.
No sustainability assessment	Food choices affect both human and planetary health.	Integration with environmental indicators.	Trade-offs between health and sustainability.
Static front-of-pack label	Limited space restricts the amount of information displayed.	Digital platforms, QR codes and AI-based decision-support tools.	Accessibility and user adoption.

Food quality dimensions considered by Nutri-Score and complementary dimensions not currently captured by nutrient profiling systems, with examples and their potential implications for health.

## Data Availability

No new data were created or analyzed in this study. Data sharing is not applicable to this article.
